# Characterization, dietary habits and nutritional intake of omnivorous, lacto-ovo vegetarian and vegan runners – a pilot study

**DOI:** 10.1186/s40795-019-0313-8

**Published:** 2019-12-03

**Authors:** Josefine Nebl, Jan Philipp Schuchardt, Paulina Wasserfurth, Sven Haufe, Julian Eigendorf, Uwe Tegtbur, Andreas Hahn

**Affiliations:** 1 Faculty of Natural Sciences, Institute of Food Science and Human Nutrition, Leibniz University Hannover, 30167 Hannover, Germany; 2 Hannover Medical School, Institute of Sports Medicine, 30625 Hannover, Germany

**Keywords:** Recreational endurance athletes, Plant-based diets; nutrient supply, Vegetarianism, Veganism, Nutrient survey

## Abstract

**Background:**

The number of people preferring plant-based nutrition is growing continuously in the western world. Vegetarianism and veganism are also becoming increasingly popular among individuals participating in sport. However, whether recreationally active vegetarian and vegan populations can meet their nutritional needs is not clear.

**Methods:**

The purpose of this cross-sectional study was to compare the nutrient intake of omnivorous (OMN, *n* = 27), lacto-ovo vegetarian (LOV, *n* = 25) and vegan (VEG, *n* = 27) recreational runners (two to five training sessions per week) with intake recommendations of the German, Austrian and Swiss Nutrition Societies (Deutsche, Österreichische und Schweizerische Gesellschaften für Ernährung, D-A-CH) for the general population. Lifestyle factors and supplement intake were examined via questionnaires; dietary habits and nutrient intake were determined based on 3-day dietary records.

**Results:**

More than half of each group did not reach the recommended energy intake (OMN: 10.4, 8.70–12.1; LOV: 9.67, 8.55–10.8; VEG: 10.2, 9.12–11.3 MJ). Carbohydrate intake was slightly below the recommendations of > 50 EN% in OMN (46.7, 43.6–49.8 EN%), while LOV (49.4, 45.5–53.3 EN%) and VEG (55.2, 51.4–59.0 EN%) consumed adequate amounts (*p* = 0.003). The recommended protein intake of 0.8 g/kg body weight (D-A-CH) was exceeded in all three groups (OMN: 1.50, 1.27–1.66; LOV: 1.34, 1.09–1.56; VEG: 1.25; 1.07–1.42 g/kg BW; *p* = 0.047). Only VEG (26.3, 22.7–29.8 EN%) did not achieve the recommended fat intake of 30 EN%. The supply of micronutrients, such as vitamin D and cobalamin, was dependent on supplement intake. Additionally, female OMN and LOV achieved the recommended daily intake of 15 mg iron only after supplementation, while VEG consumed adequate amounts solely via food.

**Conclusion:**

All three groups were sufficiently supplied with most nutrients despite the exceptions mentioned above. The VEG group even showed advantages in nutrient intake (e.g. carbohydrates, fiber and iron) in comparison to the other groups. However, the demand for energy and several macro- and micronutrients might be higher for athletes. Thus, it is also necessary to analyze the endogenous status of nutrients to evaluate the influence of a vegetarian and vegan diet on the nutrient supply of athletes.

**Trial registration:**

German Clinical Trial Register (DRKS00012377), registered on April 28, 2017.

## Background

Plant-based diets, especially vegetarianism and veganism, are increasingly gaining popularity in the western world. These alternative diets are characterized by a predominance of foodstuffs derived from plants in varying amounts and range from abstaining meat, meat products and fish to complete rejection of animal products such as vegans (VEG) [1, 2]. About 4.3 to 10% of the population in Germany are estimated to be vegetarians, whereas the number of VEG is estimated at 1.6% [3–5]. Switzerland, Italy, Austria and the UK have a similar number of vegetarians as Germany at 9–11% [6]. In the United States, only 5% of the population is considered vegetarian [7], however, this is still more than 16 million people.

It is undisputed that a lacto-ovo vegetarian (LOV) diet based on a broad variety of foods generally ensures the supply of (nearly) all nutrients in adults [1, 8, 9] and has favorable effects on the cardiometabolic risk compared to the usual mixed diet [10–14]. Moreover, plant-based diets show beneficial associations with obesity, type 2 diabetes, hypertension and cancer [15–18], although healthy omnivore (OMN) diets can achieve similar effects [19]. Consequently, several nutrition societies recommend LOV diets as a healthy diet for all stages of life [8, 20–22]. By contrast, strict VEG nutrition is viewed as critical due to the risk for an undersupply with critical nutrients such as protein, long-chain n3 fatty acids, riboflavin, cobalamin, vitamin D, calcium, iron and zinc [23]. Thorough planning and engagement with a VEG diet are required to adjust the nutrient supply and meet the needs in different population groups.

A balanced diet also plays an important role for athletes. The impact of a plant-based diet on the health and performance of athletes is becoming a growing interest [4]. However, data on the prevalence of vegetarians or VEG as recreational and professional athletes are still sparse and only a few studies have investigated the nutritional status of vegetarian athletes [24, 25, 26]. Therefore, it is of great importance to investigate the nutritional status of athletes using data on dietary habits combined with analytical data on the nutrient status and functional outcomes. Such findings enable an evaluation of whether athletes who follow plant-based diets can meet their nutritional needs or show nutrient imbalances. Furthermore, such data form the basis for assessing the relationship of a plant-based diet with the body composition, the antioxidant and immunological capacity and, ultimately, with the health and performance of athletes [24, 26, 27]. Present studies investigating the relationship between a vegetarian and VEG diet and exercise do not differentiate between vegetarians and VEG [26], are outdated [28], questionnaire-based [25, 29, 30] or do not contain nutritional assessment including biochemical markers [31, 32].

The nutrient supply status of athletes consuming a balanced mixed diet including animal-based foods can usually be classified as safe, including critical nutrients. However, there is lack of scientific data investigating the question of whether vegetarian and especially VEG athletes are undersupplied with critical nutrients, and whether this affects health and performance. To date, no data exist on the nutritional and athletic conditions of VEG recreational runners and there are no recommendations regarding nutrient intake for LOV and VEG athletes. Therefore, in order to fill the knowledge gap between nutrient intake, status and performance, the novel approach of this study is to compare the dietary habits, nutritional intake, body composition and performance diagnostics of VEG and LOV recreational runners with OMN runners. We present here a comparison of the nutritional supply status of these three groups and a comparison with reference values of the German, Austrian and Swiss Nutrition Societies for healthy adults (Deutsche, Österreichische und Schweizerische Gesellschaft für Ernährung: D-A-CH) [33]. These data may serve as a first basis to determine specific recommendations regarding the nutrient intake for vegetarian and vegan athletes in the future.

## Methods

### Participants

This cross-sectional study was conducted at the Institute of Food Science and Human Nutrition, Leibniz University Hannover, Germany. Ethical approval was provided by the Ethics Committee at the Medical Chamber of Lower Saxony (Hannover, Germany). The study was conducted in accordance with the Declaration of Helsinki. All subjects gave their written informed consent. The study was registered in the German Clinical Trial Register (DRKS00012377).

Eighty-one healthy recreational runners (mean age: 27.5 ± 4.14 yr., height: 1.75 ± 0.80 m, body mass: 67.7 ± 9.56 kg, BMI: 22.0 ± 1.94 kg/m^2^, m = 31, f = 50) aged between 18 and 35 years were recruited from the general population in Hannover, Germany, via local running events, online running communities and online vegetarian and VEG communities.

The eligibility of subjects was assessed using questionnaires. Participants were selected based on the following inclusion criteria: OMN, LOV or VEG diet for at least half a year, body mass index (BMI) between 18.5 and 25.0 kg/m^2^ and run regularly two to five times per week for at least 30–60 min. Regular running sessions were documented via self-reporting data. The following criteria led to exclusion: Any cardiovascular, metabolic or malignant disease, diseases of the gastrointestinal tract, pregnancy, food intolerances and addiction to drugs or alcohol. Participants were allowed to take dietary supplements, but the use of performance-enhancing substances (e.g. alkaline salts, creatine) led to exclusion.

### Methods and examination procedure

A questionnaire, which included food groups the participants usually consume, had to be completed to categorize subjects as OMN, LOV and VEG recreational athletes.

Participants were matched according to age and gender. Subjects who were included in the study collective were invited to an examination. Prior to the examination, subjects fulfilled a 3-day dietary record over three consecutive days, including 2 week days and one weekend day. The nutritional diaries were checked by nutritionists for completeness, readability and plausibility. Ambiguities were clarified with subjects if necessary. Seventy-nine out of 81 participants returned the completed dietary record. The following food groups were analyzed: Meat, meat products and sausages, fish and seafood, milk and dairy products, eggs, fat and oil, whole grain products, cereal products, pastries, potatoes, vegetables, legumes, soy, fresh fruits, nuts and seeds, sweets, alcoholic drinks, alcohol, nonalcoholic beverages, coffee, tea and fast food.

Nutrient intake was depicted in comparison to the reference values of the German, Austrian and Swiss Nutrition Societies for healthy adults (D-A-CH) [33]. Amino acid intake was compared to the reference values of the World Health Organization (WHO) [34].

All 81 participants completed a questionnaire regarding their supplement intake, status of health and running activity. Training frequency and duration were self-reported by the subjects. The determination of anthropometric data followed. The measurements of body weight (BW) and height were carried out without shoes, respectively. Waist circumference was determined using a tape measure. The BMI was calculated using the standard formula:

$$ BMI=\frac{\mathrm{body}\ \mathrm{mass}\ \left[\mathrm{kg}\right]}{{\left(\mathrm{height}\ \left[\mathrm{m}\right]\right)}^2} $$

### Data analysis and statistical methods

The nutrition organization software PRODI6.4® (Nutri-Science GmbH, Freiburg, Germany) was used to analyze dietary habits, energy and nutrient intake from the 3-day dietary record. The composition of foods, which were not available in PRODI6.4®, have been requested from the manufacturer and the results were integrated into the software. The intake of animal- and plant-based iron was also calculated with the software. The compositions of all supplementary products mentioned at the time of evaluation were researched and multiplied by the intake frequency (daily intake (factor *1), two times a week (factor *2/7), three times a week (factor *3/7), four times a week (factor *4/7), irregular intake (factor *12/365)) to calculate the average daily intake of the respective nutrients via supplements. Based on the intake frequencies above, the average daily intake for each mineral and vitamin was calculated for each subject individually.

Statistical analyses were performed using SPSS software (IBM SPSS Statistics 24.0; Chicago, IL, USA). Results are presented as mean ± standard deviation (SD) or 95% confidence interval (CI). Normal distribution was checked using the Kolmogorov-Smirnov test. If data were normally distributed, one-way analysis of variance (ANOVA) was used to evaluate differences in nutritional status and intake between the three diet groups. The Kruskal-Wallis test was performed to analyze data with non-normal distribution. If there were significant differences between the groups, the post hoc test with Bonferroni correction was conducted. The Mann-Whitney U test was used to examine differences between supplement users (SU) and non-supplement users (non-SU) within the groups. The chi-square test was used to compare the differences between the frequencies of the three groups. Associations between parametric data were computed via Pearson and nonparametric data via Spearman’s rho correlation. *P* values ≤0.05 were interpreted as statistically significant.

## Results

### Characterization of the study population

**Fig. 1:**
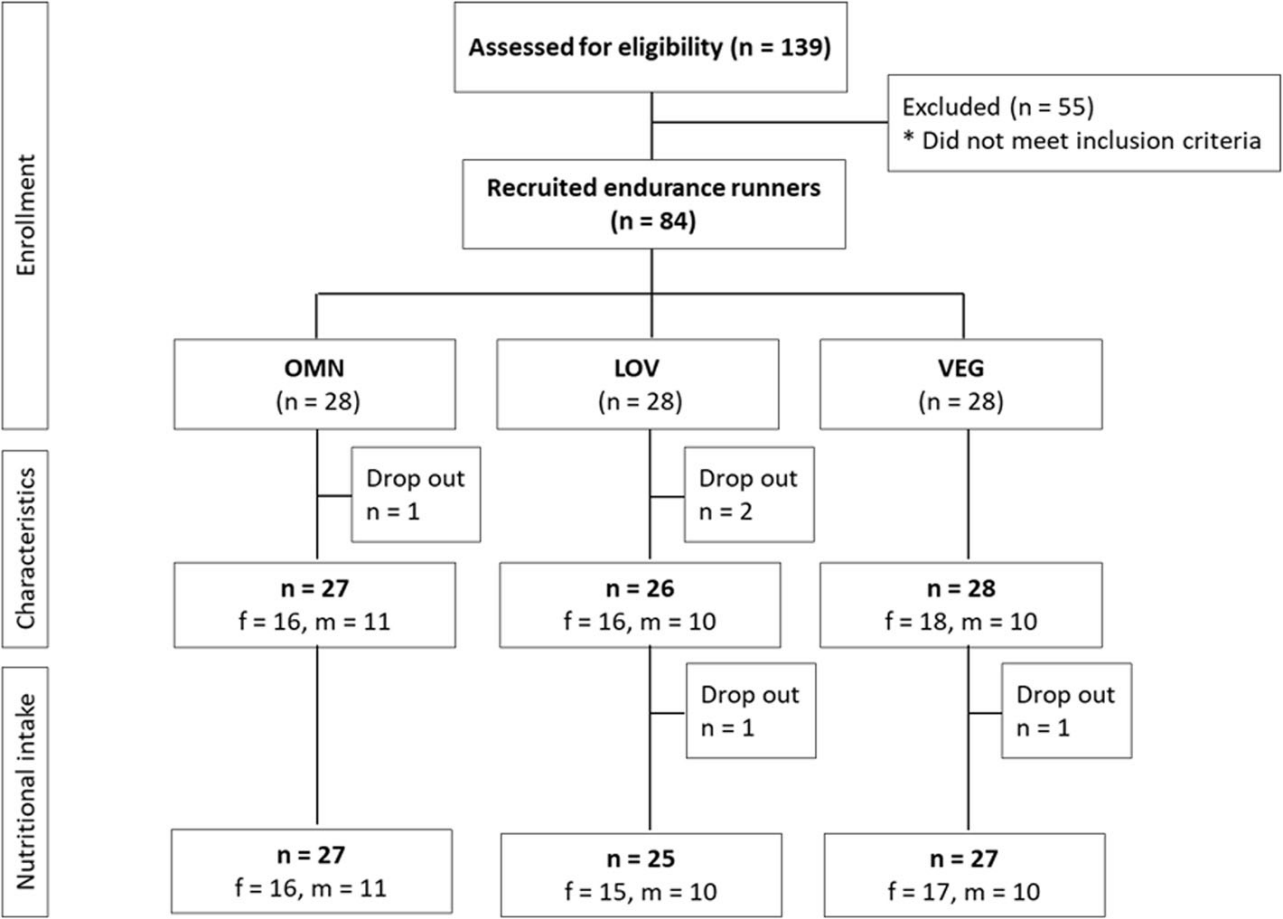
Flow chart of the study population

Twenty-seven of the 81 runners followed OMN nutrition, 26 were LOV and 28 were VEG (Fig. 1). Men and women were equally distributed and there were no differences in the mean age and anthropometric data (Table 1). Only one female of the LOV had a waist circumference slightly over 80 cm; all other participants had values in the reference range of < 80 cm for women and < 94 cm for men. All but one of the 27 participants of the OMN group had followed the diet for > 3 years. By contrast, 4 out of 26 participants of the LOV group and 6 out of 28 of the VEG group had switched to their current diet between 0.5–1 year.

**Table 1:** Characterization of the study population (mean ± SD)

	OMN (*n* = 27)	*p* value OMN-LOV	LOV (*n* = 26)	*p* value LOV-VEG	VEG (*n* = 28)	*p* value OMN-VEG	*p* value 3 groups
Age, years	27.4 ± 4.03	–	27.6 ± 4.31	–	27.5 ± 4.24	–	0.968^b^
Sex	m = 11, w = 16	–	m = 10, w = 16	–	m = 10, w = 18	–	0.929^c^
BMI, kg/m^2^	22.3 ± 1.74	–	21.6 ± 1.98	–	22.1 ± 2.09	–	0.436^b^
Waist, cm							
Female	71.0 ± 4.3	-	70.1 ± 3.8	-	69.5 ± 5.0	-	0.057^a^
Male	79.5 ± 4.3	-	76.4 ± 3.0	-	80.6 ± 4.1	-	0.591^a^
Systolic blood pressure, mm Hg	121 ± 11.1	–	121 ± 13.4	–	116 ± 12.6	–	0.201^b^
Diastolic blood pressure, mm Hg	74.0 ± 6.00	–	72.0 ± 4.00	–	72.0 ± 9.00	–	0.457^b^
Pulse rate, bpm	66.0 ± 9.00	–	61.0 ± 8.00	–	65.0 ± 10.00	–	0.188^b^
Duration of diet							**0.001**^**c**^
< 0.5 years, n (%)	0 (0)		0 (0)		0 (0)		
0.5–1 year, n (%)	0 (0)		4 (15.4)		6 (21.4)		
1–2 years, n (%)	1 (3.7)		3 (11.5)		4 (14.3)		
2–3 years, n (%)	0 (0)		2 (7.7)		7 (25.0)		
> 3 years, n (%)	26 (96.3)		17 (65.4)		11 (39.3)		
Magnesium SU, n (%)	5 (22.2)		4 (15.4)		5 (17.9)		0.710^c^
Calcium SU, n (%)	3 (11.1)		1 (3.9)		2 (7.14)		0.210^c^
Iron SU, n (%)	3 (11.1)		4 (15.4)		5 (17.9)		0.689^c^
Vitamin B_12_ SU, n (%)	4 (18.5)		4 (15.4)		15 (53.9)		**0.005**^**c**^
Vitamin D SU, n (%)	5 (22.2)		1 (3.9)		7 (25.0)		0.078^c^
Smoker, n (%)	0 (0)		0 (0)	–	0 (0)	–	–
Training frequency per week	3.04 ± 0.98	–	3.24 ± 0.88	–	3.00 ± 0.85	–	0.502^b^
Running time per week (h)	2.72 ± 1.11	**–**	3.38 ± 1.43	–	2.65 ± 1.38	–	0.079^b^

*OMN* omnivores, *LOV* lacto-ovo vegetarians, *VEG* vegans, *BMI* body mass index, *bpm* beats per minute, *SU* supplement users

^a^One-way ANOVA, ^b^Kruskal Wallis test, ^c^Chi-square test, *p*-values ​​in bold represent statistical significance

**Fig. 2:**
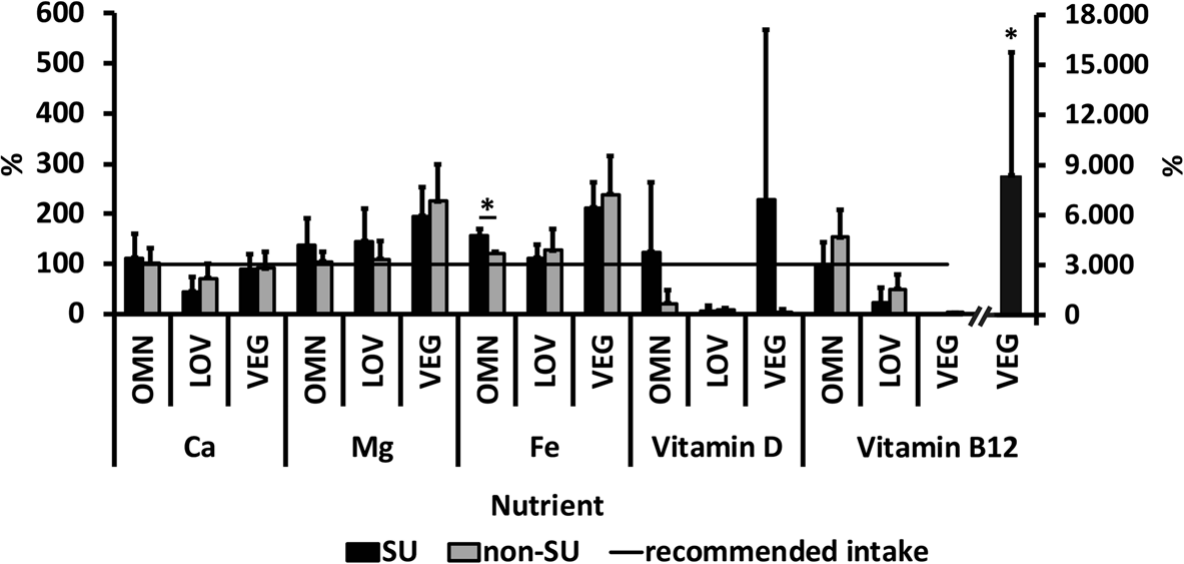
Nutrient intake in relation to the reference range: Supplement users vs. non-supplement users (males; mean + SD). OMN = omnivores, LOV = lacto-ovo vegetarians, VEG = vegans, SU = supplement users, non-SU = non-supplement users, recommended intake of the German, Austrian and Swiss Nutrition Societies (Deutsche, Österreichische und Schweizerische Gesellschaften für Ernährung, D-A-CH) [33]. The error bars represent the standard deviations of the average daily nutrient intake. Differences between SU and non-SU were analyzed using the Mann-Whitney U test. *p ≤ 0.05

**Fig. 3:**
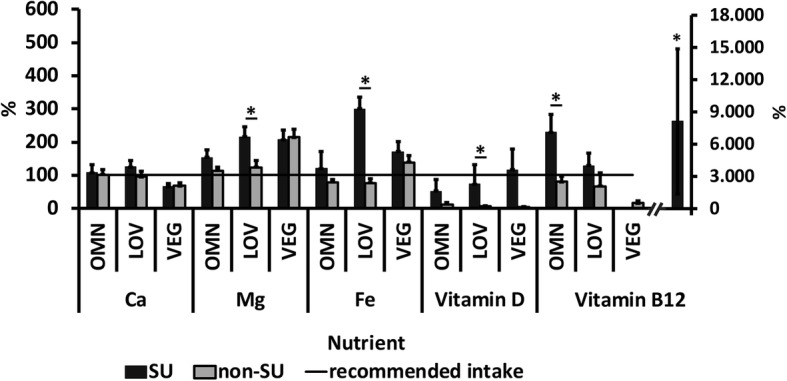
Nutrient intake in relation to the reference range: SU vs. non-SU (females; mean + SD). Recommended intake of the D-A-CH [33]. The error bars represent the standard deviations of the average daily nutrient intake. Differences between SU and non-SU were analyzed using the Mann-Whitney U test. *p ≤ 0.05

Several subjects took dietary supplements. More precisely, 18 out of 28 participants (64.3%) of the VEG, 10 out of 27 (37.0%) of the OMN and 9 out of 26 (34.6%) of the LOV group took supplements. Although considerably more subjects of the VEG group consumed supplements, there were no statistically significant differences between the groups. Magnesium, calcium, iron, cobalamin and vitamin D were commonly consumed supplements (Table 1). Magnesium and vitamin D were most commonly supplemented in the OMN group (22.2% and not significant [n.s.], respectively), magnesium, iron and cobalamin in LOV (15.4%; n.s.), and cobalamin in VEG (53.9%; *p* = 0.005, χ^2^). Total nutrient intake of SU compared to non-SU was investigated (Figs. 2 and 3). Statistically significantly higher cobalamin intake in SU compared to non-SU was found in both male and female VEG (*p* = 0.019 and 0.003, respectively) as well as in female OMN (*p* = 0.027) and LOV (*p* = 0.026). Magnesium (*p* = 0.036), vitamin D (*p* = 0.018) and iron (*p* = 0.018) intake was statistically significantly higher in female LOV SU compared to non-SU. Male SU in OMN also showed higher iron intakes than non-SU (*p* = 0.004). The analysis of fortified food products revealed only one subject who consumed a small amount (15 mg) of calcium-enriched soy drink, which can be neglected.

None of the subjects regularly consumed tobacco. The participants showed no differences in training frequency or duration (Table 1).

### Dietary habits

According to their diet, LOV and VEG consumed neither meat, meat products, fish nor seafood (Table 2). The VEG additionally waived milk, dairy products and eggs. The three groups consumed similar amounts of fat and oil, whole grain and cereal products as well as pastries. Moreover, there were no significant differences in the dietary intake of sweets, alcoholic drinks, coffee and tea. The VEG consumed significantly higher amounts of potatoes, vegetables and fresh fruit compared to LOV (p_LOV-VEG_ = 0.013, 0.031 and 0.041, respectively) and OMN (p_OMN-VEG_ = 0.017, 0.000 and 0.015, respectively). Legumes were consumed mainly in the VEG group (*p* < 0.001), while OMN consumed the highest amounts of fast food (*p* = 0.016) (Table 2).

**Table 2:** Mean daily intake of different food categories calculated from a 3-day dietary record

Food group (g/day)	OMN (*n* = 27)	*p* value OMN-LOV	LOV (*n* = 25)	*p* value LOV-VEG	VEG (*n* = 27)	*p* value OMN-VEG	*p* value 3 groups
Meat	85.8 ± 58.8	0.000^c^	–	1.000^c^	–	0.000^c^	**0.000**^**b**^
Meat products and sausages	29.6 ± 32.1	0.000^c^	–	1.000^c^	–	0.000^c^	**0.000**^**b**^
Fish and seafood	28.7 ± 39.9	0.000^c^	–	1.000^c^	–	0.000^c^	**0.000**^**b**^
Milk and dairy products	290 ± 183	1.000^c^	279 ± 311	0.000^c^	–	0.000^c^	**0.000**^**b**^
Eggs	23.8 ± 37.4	1.000^c^	15.8 ± 25.0	0.003^c^	–	0.000^c^	**0.000**^**b**^
Fat and oil	9.85 ± 14.8	–	10.3 ± 12.1	–	12.0 ± 10.8	–	0.228^b^
Whole grain products	33.2 ± 48.7	–	50.6 ± 58.8	–	51.0 ± 59.0	–	0.294^b^
Cereal products	208 ± 141	–	188 ± 130	–	220 ± 120	–	0.678^a^
Pastries	58.8 ± 50.0	–	58.0 ± 100	–	37.4 ± 73.8	–	0.067^b^
Potatoes	44.1 ± 79.3	1.000^c^	37.5 ± 62.3	0.013^c^	118 ± 130	0.017^c^	**0.005**^**b**^
Vegetables (except potatoes, legumes)	265 ± 237	0.511^c^	324 ± 187	0.031^c^	521 ± 258	0.000^c^	**0.000**^**b**^
Legumes (except soybeans)	3.70 ± 8.08	0.054^c^	27.7 ± 39.7	0.092^c^	66.4 ± 68.1	0.000^c^	**0.000**^**b**^
Soybeans	–	0.007^c^	54.4 ± 95	0.031^c^	151 ± 179	0.000^c^	**0.000**^**b**^
Fresh fruit	266 ± 160	1.000^c^	288 ± 171	0.041^c^	518 ± 404	0.015^c^	**0.009**^**b**^
Nuts and seeds	4.57 ± 8.30	0.044^c^	19.7 ± 23.7	0.578^c^	26.0 ± 29.3	0.000^b^	**0.001**^**b**^
Sweets	37.0 ± 39.3	–	38.9 ± 44.4	–	20.2 ± 33.6	–	0.148^b^
Alcoholic drinks	131 ± 210	–	101 ± 198	–	63.0 ± 146	–	0.184^b^
Alcohol	5.50 ± 8.64	–	3.89 ± 6.91	–	2.26 ± 5.57	–	0.345^b^
Nonalcoholic beverages (except coffee and tea)	1103 ± 1095	–	794 ± 1098	–	1246 ± 1258	–	0.339^b^
Coffee	170 ± 164	–	279 ± 238	–	148 ± 198	–	0.051^b^
Tea	257 ± 398	–	181 ± 310	–	221 ± 339	–	0.999^b^
Fast food	57.1 ± 75.2	0.063^c^	32.7 ± 87.2	1.000^c^	16.6 ± 38.1	0.025^c^	**0.016**^**b**^

All nutrients excluding dietary supplements. *OMN* omnivores, *LOV* lacto-ovo vegetarians, *VEG* vegans

Data are presented as mean ± SD. ^a^One-way ANOVA, ^b^Kruskal Wallis test, ^c^Post hoc test, *p*-values ​​in bold represent statistical significance

### Nutritional intake

None of the three groups differed in terms of energy consumption (Table 3); men (OMN: 12.3, 8.36–16.1; LOV: 10.3, 8.96–11.7; VEG: 11.5, 8.97–1 3.9 MJ; n.s.) had a higher energy intake than women (OMN: 9.11, 7.96–10.3; LOV: 9.22, 7.51–10.9; VEG: 9.47, 8.47–10.4 MJ; n.s.), which was statistically significant for OMN (*p* = 0.023). In comparison to the recommended values for people who perform sport several times a week (age group 19–25 and 25–51, physical activity level was estimated at 1.7 [33];), only the average of female VEG and male OMN reached the recommendations. Low levels of energy intake were evident in 59.3% of OMN, 52.0% of LOV and 51.9% of VEG, with no differences in frequency distribution. No significant associations were found between energy intake and age, BMI and frequency of training.

**Table 3:** Absolute and relative daily energy and macronutrient intake of the study population calculated from a 3-day dietary record

Nutrient intake	OMN (*n* = 27)	*p* value OMN-LOV	LOV (*n* = 25)	*p* value LOV-VEG	VEG (*n* = 27)	*p* value OMN-VEG	*p* value 3 groups	Reference values (m/f)*
Energy								
Energy intake (MJ)	10.4 (8.70, 12.1)	–	9.67 (8.55, 10.8)	–	10.2 (9.12, 11.3)	–	0.989^b^	11.9–12.3/9.41–9.83
Macronutrients								
Carbohydrate (EN%)	46.7 (43.6, 49.8)	0.824^c^	49.4 (45.5, 53.3)	0.067^c^	55.2 (51.4, 59.0)	0.002^c^	**0.003**^**a**^	> 50
Carbohydrate (g/kg BW)	4.31 (3.45, 5.17)	1.000^c^	4.22 (3.52, 4.91)	0.094^c^	5.01 (4.40, 5.62)	0.111^c^	**0.049**^**b**^	
Protein (EN%)	16.7 (15.1, 18.9)	0.540^c^	15.9 (13.6, 18.2)	0.295^c^	13.8 (12.5, 15.0)	0.007^c^	**0.009**^**b**^	
Protein (g/kg BW)	1.50 (1.27, 1.66)	0.159^c^	1.34 (1.09, 1.56)	1.000^c^	1.25 (1.07, 1.42)	0.063^c^	**0.047**^**b**^	0.8
Fat (EN%)	32.5 (30.5, 34.5)	0.432^c^	30.8 (26.8, 34.8)	0.708^c^	26.3 (22.7, 29.8)	0.021^c^	**0.026**^**b**^	30
Fiber (g)	27.0 (22.8, 31.1)	0.176^c^	33.4 (28.6, 38.2)	0.006^c^	51.7 (44.1, 59.4)	0.000^c^	**0.000**^**b**^	≥ 30

*OMN* omnivores, *LOV* lacto-ovo vegetarians, *VEG* vegans, *MJ* mega joule, *EN%* energy percent, *BW* body weight, *Reference values of the German, Austrian and Swiss Nutrition Societies (Deutsche, Österreichische und Schweizerische Gesellschaften für Ernährung, D-A-CH) [33]. Data are presented as mean (95% CI). ^a^One-way ANOVA, ^b^Kruskal Wallis test, ^c^Post hoc test, *p*-values ​​in bold represent statistical significance

Regarding the **macronutrient** intake, there were significant differences between OMN and VEG. The VEG consumed a higher percentage of carbohydrates (55.2, 51.4–59.0 energy percent, EN%) compared to OMN (46.7, 43.6–49.8 EN%; p_OMN-VEG_ = 0.002) (Table 3). Most subjects of the OMN group (70.4%) and 50.2% of the LOV group had low levels (< 50 EN%) of carbohydrates. By contrast, most subjects (66.7%) of the VEG group had higher levels of carbohydrates (> 55 EN%). These differences were statistically significant (*p* = 0.035, χ^2^). The absolute intake of carbohydrates differed only slightly.

Regarding the absolute dietary protein intake, there were only minor differences between the groups (Table 3). On average, all the groups were above the reference value of 0.8 g/kg BW; only one subject of the OMN group (3.70%), two subjects of LOV (8.00%) and two subjects of VEG (7.41%) did not reach the recommendations (data not shown). All three groups were adequately supplied with all essential amino acids (see Additional file 1).

Considering the average relative fat intake, subjects in the OMN group (p_OMN-VEG_ = 0.021) and LOV (n.s. compared to VEG) consumed higher amounts compared to VEG, who were below the recommendation of 30 EN% (Table 3). A low-fat intake (< 30 EN%) was observed in 70.4% of the VEG, 44.0% of the LOV and 25.9% of the OMN group. These differences were significant (*p* = 0.004, χ^2^). Differences in fatty acid intake patterns were observed. The highest intake of saturated fatty acids was observed in the OMN group (8.70, 7.13–10.3 EN%) followed by LOV (7.86, 6.17–9.55 EN%; n.s. compared to OMN) and VEG (4.57, 3.55–5.59 EN%; p_OMN-VEG_ < 0.001) (see Additional file 2). Monounsaturated fatty acids were least consumed by the VEG group (3.96, 3.02–4.91 EN%) compared to LOV (5.45, 3.77–7.13 EN%; n.s. compared to LOV) and OMN (5.95, 4.86–7.03 EN%; p_OMN-VEG_ = 0.019). No differences were observed in polyunsaturated fatty acid (PUFA) intake. On average, none of the three groups reached the recommended intake values of monounsaturated fatty acids (> 10 EN%) and PUFA (7–10 EN%). The intake of linoleic acid (LA) was 4.33 (3.44–5.21) EN% in the VEG group, 3.52 (2.57–4.46) EN% in LOV and 2.96 (2.50–3.42) EN% in OMN. Similarly, the intake of alpha-linolenic acid (ALA) was highest in the VEG group (0.80, 0.55–1.05 EN%) compared to LOV (0.68, 0.33–1.03 EN%, n.s. compared to VEG) and OMN (0.37, 0.27–0.48 EN%, p_OMN-VEG_ = 0.005). The ratio LA:ALA did not differ significantly between the groups, although OMN showed a less favorable ratio (1:8.04) (see Additional file 2). The PUFAs, eicosapentaenoic acid (EPA, 20:5n3) and docosahexaenoic acid (DHA, 22:6n3), were supplemented by two subjects of the OMN group, two subjects of the VEG and one of the LOV group. We observed the highest sum of EPA + DHA intake in the OMN group (0.54, 0.23–0.85 g), followed by LOV (0.08, 0.37–0.12 g; p_OMN-LOV_ = 0.003) and VEG (0.09, 0.01–0.17 g; p_OMN-VEG_ < 0.001).

Fiber intake was significantly higher in the VEG group (51.7, 44.1–59.4 g) compared to LOV (33.4, 28.6–38.2 g; p_OMN-LOV_ = 0.006) and OMN (27.0, 22.8–31.1 g; p_OMN-VEG_ < 0.001). The latter did not reach the minimum reference value of 30 g per day.

**Micronutrient** intakes also showed several differences between the groups (Table 4). Several participants did not reach the recommended intake for all the micronutrients examined (see Additional file 3). There were variations regarding the minerals sodium, potassium and magnesium, while calcium and phosphorus values were similar. More precisely, lower sodium intake was observed in LOV (p_OMN-LOV_ = 0.004) and VEG (p_OMN-VEG_ = 0.005) compared to OMN (Table 4, *p* values of total intake are not shown). By contrast, the VEG group had significantly higher intake levels of potassium and magnesium compared to LOV (p_LOV-VEG_ = 0.005 and 0.001, respectively) and OMN (p_OMN-VEG_ = 0.014 and < 0.001, respectively) (Table 4, p values of total intake are not shown). On average, the LOV and VEG groups had calcium intakes < 1000 mg per day [33], and OMN consumed sufficient amounts (1026, 846–1207 mg) due to supplementation. A total of 64.0% of the LOV group, 51.9% of OMN and 44.4% of VEG were below the recommendations for calcium (see Additional file 3).

**Table 4:** Dietary mineral intake of the study population calculated from a 3-day dietary record (nutrient intake via food and supplements)

	OMN (*n* = 27)	*p* value OMN-LOV	LOV (*n* = 25)	*p* value LOV-VEG	VEG (*n* = 27)	*p* value OMN-VEG	*p* value 3 groups	Reference values (m/f)*
Na (g)								
Food	2.65 (2.17, 3.12)	0.004^b^	1.72 (1.44, 2.00)	1.000^b^	1.72 (1.46, 1.99)	0.005^b^	**0.001**^**a**^	1.5
Supplement	0	–	0	–	0	–	–	
K (g)								
Food	3.16 (2.88, 3.50)	1.000^b^	3.04 (2.55, 3.52)	0.005^b^	4.65 (3.85, 5.50)	0.014^b^	**0.002**^**a**^	4^c^
Supplement	0	–	0.00 (0.00, 0.01)	–	0.00 (0.00, 0.01)	–	0.372^a^	
Ca (mg)								
Food	981 (813, 1149)	–	901 (716, 1085)	–	730 (614, 846)	–	0.115^a^	1000
Supplement	45.1 (−32.0, 122)	–	0	–	6.37 (−2.22, 15.0)	–	0.214^a^	
P (g)								
Food	1.43 (1.26, 1.60)	–	1.34 (1.08, 1.61)	–	1.33 (1.15, 1.52)	–	0.495^a^	0.7
Supplement	0	–	0	–	0	–	–	
Mg (mg)								
Food	346 (310, 382)	0.990^b^	388 (324, 452)	0.001^b^	599 (518, 679)	0.000^b^	**0.000**^**a**^	350/300
Supplement	36.7 (0.44, 73.0)	–	53.2 (−5.58, 112)	–	54.3 (−7.09, 116)	–	0.910^a^	
Fe (mg)								
Food (total)	11.9 (10.6, 13.2)	1.000^b^	12.8 (10.8, 14.7)	0.001^b^	19.6 (16.8, 22.4)	0.000^b^	**0.000**^**a**^	10/15
Plant-based iron	7.44 (6.33, 8.54)	0.105^b^	10.7 (8.95, 12.5)	0.000^b^	19.6 (16.8, 22.4)	0.000^b^	**0.000**^**a**^	
Animal iron	4.45 (3.67, 5.24)	0.013^b^	2.02 (1.41, 2.61)	0.000^b^	0	0.000^b^	**0.000**^**a**^	
Supplement	1.70 (−1.36, 4.77)	–	1.52 (−1.19, 4.24)	–	3.74 (−0.64, 8.12)	–	0.675^a^	
Zn (mg)								
Food	9.74 (8.32, 11.2)	–	8.88 (7.30, 10.5)	–	10.7 (9.21, 12.2)	–	0.214^a^	14/8^1^
Supplement	2.23 (− 1.59–6.04)	–	0.90 (−0.70–2.49)	–	0.47 (− 0.48–1.41)	–	0.648^a^	
Cu (mg)								
Food	1.63 (1.43, 1.84)	0.819^b^	1.85 (1.56, 2.13)	0.001^b^	2.93 (2.51, 3.34)	0.000^b^	**0.000**^**a**^	1.0–1.5
Supplement	0	–	0	–	0	–	**–**	
Mn (mg)								
Food	4.75 (3.87, 5.62)	0.188^b^	6.29 (5.05, 7.54)	0.067^b^	8.48 (7.10, 9.85)	0.000^b^	**0.000**^**a**^	2.0–5.0
Supplement	0	–	0	–	0	–	**–**	
I (μg)								
Food	88.8 (64.1, 114)	0.190^b^	61.6 (49.4, 73.7)	1.000^b^	57.7 (48.4, 67.0)	0.060^b^	**0.048**^**a**^	200
Supplement	0	–	0	–	0	–	**–**	

*OMN* omnivores, *LOV* lacto-ovo vegetarians, *VEG* vegans, *Reference values of the German, Austrian and Swiss Nutrition Societies (Deutsche, Österreichische und Schweizerische Gesellschaften für Ernährung, D-A-CH) [33]

Data are presented as mean (95% CI). ^a^ Kruskal Wallis test, ^b^ Post hoc test, ^c^ Estimated values, *p*-values ​​in bold represent statistical significance ^1^ At medium phytate intake

There were also group differences regarding trace elements, except for the zinc values, which did not vary between the groups. All three groups had adequate dietary zinc intakes, however, the male LOVs were slightly low (9.89, 5.33–14.5 mg). Female subjects reached the recommendations and so did the non-SU (OMN: 8.46, 6.30–10.6 mg; LOV: 9.44, 6.77–12.1 mg; VEG: 9.89, 7.63–12.1 mg). We observed a high iron intake, particularly in the VEG group (Table 4). The mean iron intake was within the recommended area (10 mg/day [33]) in all three groups when only men were compared, and in both male SU and non-SU (Fig. 2). The highest iron intake via food in women was found in the VEG group (19.8, 15.7–24.0 μg), followed by LOV (12.8, 9.47–16.1 μg; p_LOV-VEG_ = 0.037) and OMN (11.2, 9.01–13.2 μg; p_OMN-VEG_ = 0.005). Only the female SU in both the LOV and OMN groups reached the reference range (15 mg/day [33]) (Fig. 3). The iron sources in the diet of the VEG group were exclusively plant-based food. However, the LOV and OMN groups consumed predominantly plant-based iron as well (Table 4). The worst supply was observed for iodine. Only 3.7% of the OMN group and none of the subjects in LOV and VEG had values in a reference range of 200 μg per day (see Additional file 3) [33].

Variations were also observed in the vitamin intake between the groups (Table 5). On average, all three groups reached the recommended amounts for thiamine, pyridoxine and folate, while the reference value for vitamin D was not achieved, and the ascorbic acid intake was exceeded in all groups. Due to the supplementation, the highest average intake of cobalamin was observed in the VEG group (207, 102–313 μg), followed by OMN (4.97, 3.70–6.25 μg; n.s. compared to VEG) and LOV (2.96, 1.69–4.24 μg; n.s. compared to VEG) (Table 5). Riboflavin intake was low in 44.4% of VEG subjects, 44.0% of LOV and 22.2% of OMN (see Additional file 3). We found the highest vitamin D intake in the VEG group (19.9, 2.75–37.0 μg), followed by OMN (8.29, 2.22–14.37 μg; n.s. compared to VEG) and LOV (4.52, − 1.34–10.39 μg; n.s. compared to VEG) (Table 5). Only 22.2% of the VEG group, 14.8% of OMN and 4.00% of LOV had vitamin D intakes within the recommendations (20 μg/day [33]).

**Table 5:** Dietary vitamin intake of the study population calculated from a 3-day dietary record (nutrient intake via food and supplements)

		OMN (*n* = 27)	*p* value OMN-LOV	LOV (*n* = 25)	*p* value LOV-VEG	VEG (*n* = 27)	*p* value OMN-VEG	*p* value 3 groups	Reference values (m/f)*
A [retinol equ.] (mg)	Food	1.45 (0.81, 2.10)	–	1.26 (0.91, 1.61)	–	1.72 (1.27, 2.16)	–	0.221^a^	1.0/0.8
	Supplement	0	–	0	–	0	–	–	
D (μg)	Food	2.61 (1.34, 3.89)	1.000^b^	1.67 (1.02, 2.32)	0.037^b^	1.04 (0.46, 1.62)	0.003^b^	**0.002**^**a**^	20
	Supplement	5.68 (−0.12, 11.5)	–	2.75 (−2.91, 8.40)	–	18.8 (1.61, 36.1)	–	0.086^a^	
E (mg)	Food	9.66 (7.85, 11.5)	0.851^b^	11.4 (9.03, 13.7)	0.280^b^	16.4 (12.5, 20.4)	0.015^b^	**0.018**^**a**^	14/12^c^
	Supplement	1.12 (−0.47, 2.71)	–	0.15 (− 0.16, 0.47)	–	0.04 (− 0.37, 0.11)	–	0.411^a^	
K (μg)	Food	92.5 (63.5, 122)	0.119^b^	181 (96.6, 266)	0.058^b^	261 (164, 359)	0.000^b^	**0.000**^**a**^	70/60
	Supplement	0	–	0	–	0	–	**–**	
B_1_ [thiamine] (mg)	Food	1.38 (1.21, 1.55)	0.502^b^	1.20 (0.98, 1.43)	0.003^b^	1.86 (1.56, 2.16)	0.143^b^	**0.004**^**a**^	1.2/1.0
	Supplement	0.56 (− 0.58, 1.70)	–	0.17 (−0.16, 0.50)	–	0.09 (− 0.08, 0.26)	–	0.888^a^	
B_2_ [riboflavin] (mg)	Food	1.57 (1.34, 1.79)	–	1.54 (1.12, 1.96)	–	1.38 (1.16, 1.59)	–	0.278^a^	1.4/1.1
	Supplement	0.56 (−0.58, 1.70)	–	0.01 (− 0.01, 0.03)	–	0.11 (− 0.98, 0.33)	–	0.896^a^	
Niacin (mg)	Food	21.4 (18.5, 24.3)	0.033^b^	15.8 (12.3, 19.3)	1.000^b^	17.3 (12.3, 22.3)	0.021^b^	**0.010**^**a**^	15/12
	Supplement	0.62 (−0.52, 1.77)	–	0.09 (−0.09, 0.27)	–	1.31 (− 1.12, 3.74)	–	0.645^a^	
Pantothenic acid (mg)	Food	5.23 (4.38, 6.07)	–	5.36 (4.04, 6.68)	–	6.39 (4.96, 7.81)	–	0.461^a^	6^c^
	Supplement	0.95 (− 0.95, 2.85)	–	0	–	0.04 (− 0.19, 0.11)	–	0.374^a^	
B_6_ [pyridoxine] (mg)	Food	1.91 (1.61, 2.20)	0.670^b^	1.59 (1.27, 1.91)	0.002^b^	2.63 (2.10, 3.16)	0.087^b^	**0.003**^**a**^	1.6/1.4
	Supplement	0.47 (− 0.31, 1.25)	–	0.46 (− 0.11, 1.04)	–	0.16 (− 0.07, 0.40)	–	0.497^a^	
Biotin (μg)	Food	50.9 (44.9, 56.9)	–	56.7 (43.4, 69.9)	–	64.5 (51.4, 77.6)	–	0.573^a^	30–60^c^
	Supplement	6.10 (−5.33, 17.5)	–	0	–	0.70 (−0.44, 1.90)	–	0.373^a^	
Folate (μg)	Food	307 (249, 364)	1.000^b^	327 (265, 389)	0.024^b^	478 (402, 572)	0.001^b^	**0.001**^**a**^	300
	Supplement	11.3 (−5.01, 27.6)	–	2.20 (−2.33, 6.72)	–	41.9 (−20.2, 104)	–	0.261^a^	
B_12_ [cobalamin] (μg)	Food	4.02 (3.12, 4.92)	0.057^c^	2.49 (1.49, 3.48)	0.002^b^	0.79 (0.47, 1.12)	0.000^b^	**0.000**^**a**^	4
	Supplement	0.96 (−0.21, 2.13)	0.002^b^	0.84 (−0.20, 1.89)	1.000^b^	206 (101, 312)	0.004^b^	**0.001**^**a**^	
C [ascorbic acid] (mg)	Food	153 (110, 196)	1.000^b^	143 (107, 179)	0.003^b^	293 (222, 365)	0.001^b^	**0.000**^**a**^	110/95
	Supplement	3.16 (−1.07, 7.38)	–	0.17 (−0.18, 0.51)	–	7.80 (−1.26, 13.7)	–	0.126^a^	

*OMN* omnivores, *LOV* lacto-ovo vegetarians, *VEG* vegans, *retinol equ*. retinol equivalent, *Reference values of the German, Austrian and Swiss Nutrition Societies (Deutsche, Österreichische und Schweizerische Gesellschaften für Ernährung, D-A-CH) [33]

Data are presented as mean (95% CI). ^a^Kruskal Wallis test, ^b^Post hoc test, ^c^Estimated values, *p*-values ​​in bold represent statistical significance

## Discussion

Organizations such as *The American College of Sports Medicine* (ACSM), *The International Society for Sports Nutrition* (ISSN) and the *International Olympic Committee* (IOC) have defined guidelines for athletes [35–37]. As these few existing recommendations for mainly high-performance athletes were only partially applicable to this study collective, the nutrient intake was compared with intake recommendations of the D-A-CH for the general population. However, the D-A-CH does not specify any certain reference values for ambitious recreational athletes [33].

In general, recreational athletes can be supplied with all micronutrients through a balanced mixed diet. But, it is unknown whether a vegetarian and especially vegan diet can provide all the important nutrients for athletes.

The type, duration and intensity of sport determines the energy requirements. The ISSN recommends an energy intake from 7.5–10.0 MJ (1800–2400 kcal) for athletes with general physical activity levels of 30–40 min three to four times a week [35]. In order to assess the energy demand, the ACSM recommends various options (e.g. based on the daily recommended intake, the basal metabolic rate and a factor of physical activity or metabolic equivalents) [37]. The IOC refers to the fat-free mass (30–45 kcal/kg FFM/day) [38]. Our subjects trained an average of three times a week for about 60 min, which corresponds to an estimated physical activity level value of about 1.7 (sedentary work and recreationally active) [33]. More than half of each group did not reach the recommended energy intake, which is not uncommon in endurance athletes [39]. There were no differences among the groups, which agrees with the results of Lynch and colleagues, who compared 35 vegetarian athletes with 35 omnivores [26].

### Macronutrients

Carbohydrates are the most important sources of energy and many endurance athletes strive to consume carbohydrates to benefit from full glycogen stores [40]. Depending on the intensity and type of training or competition, gender, and external influences, an absolute amount of 3–7 g/kg BW is recommended for people with general physical activity of about 30–60 min/day 3–4 times a week up to about 1 hour a day [35–37]. Thus, participants in the present study achieved the recommendations for carbohydrate intake [35–37]. Similar to previous studies with non-athletes [41–44], the VEG group had the highest intake of carbohydrates (55.2, 51.4–59.0 EN%) compared to OMN (46.7, 43.6–49.8 EN%; p_OMN-VEG_ = 0.002) and LOV (49.4, 45.5–53.3 EN%; n.s. compared to VEG), which can be explained by the increased intake of potatoes and fruit, since the intake of whole-grain and cereal products, pastries and sweets were similar for all groups.

The protein needs of athletes have been widely discussed [45–47]. The three societies recommend a range of 1.2–2.0 g/kg BW for most exercising individuals (including general fitness [35]) [35–37]. According to the IOC and ACSM, the recommended amount also applies to vegetarians. The average protein intake of all three groups was within the reference range. In addition to absolute protein intake, it is important to consider the quality of the proteins [35]. Protein sources were mainly meat, meat products and sausages, fish and dairy products for the OMN group, milk, dairy products, and eggs for LOV, and cereal products, legumes and soybeans for VEG. In general, a high biological value can be achieved with each of these three diets. Compared to the reference values of the WHO, on average, all groups met the reference range for amino acid intake [34]. Hence, it can be assumed that all three groups – including VEG – had an adequate protein and amino acid supply. Our findings are consistent with the literature, which has shown that non-athlete LOV and VEG appear to be within the range of recommendations for protein intake [44, 48].

Dietary fats are valuable energy sources and have structural and regulatory functions. Dietary recommendations for adequate fat intakes vary widely and depend on the level of training and body composition goals [35–37]. While the ACSM recommends a daily intake of 20–35 EN% but not less than 20 EN% fat [37], the IOC advises an intake of ≥15–20 EN% fat, depending on the type of sport [49]. By contrast, both D-A-CH and ISSN recommend a fat intake of 30 EN% [33, 35]. Most subjects in the three groups reached the recommendations of the D-A-CH [33], ISSN and ACSM. In addition, it is important to evaluate the PUFA intake of athletes, which was below the reference value in all three groups [33]. PUFAs play a pivotal role in health due to their precursor function as regulatory lipid mediators. The International Society for the Study of Fatty Acids and Lipids recommends a daily sum EPA + DHA intake of 0.5 g, which was achieved by the OMN group (0.54, 0.23–0.85 g), but not by LOV (0.08, 0.04–0.12 g; p_OMN-LOV_ = 0.003) or VEG (0.09, 0.01–0.17 g; p_OMN-VEG_ < 0.001) [50]. PUFA intakes in LOV and VEG within this study can be classified as inadequate, which is consistent with other studies regarding non-athlete vegetarians and vegans [51]. The EPA/DHA supplements were only consumed occasionally in the VEG and LOV groups. The resulting LA:ALA ratios in the VEG (1:5.71) and LOV groups (1:5.30) were within the reference range [33]. The OMN group showed higher LA:ALA ratios (1:8.04), which are consistent with the results of the German Nutrition Survey [52].

### Micronutrients

It is generally thought that athletes consume high amounts of micronutrients via dietary supplements due to their increased health awareness [53]. However, several studies have shown insufficient micronutrient intake in athletes [54, 55]. There are no specific recommendations for micronutrient intakes in recreationally active individuals, which differ from the general population’s guidelines. However, in the view of the ACSM, ISSN and IOC, an adequate supply of micronutrients is assured with a balanced mixed diet. A possible insufficient supply to vegetarians of zinc, iron, riboflavin, cobalamin and vitamin D is described in the ACSM and IOC guidelines [36, 37], while the ACSM additionally mentions calcium, pyridoxine and folate. A specific risk of an insufficient micronutrient supply with a vegan diet is not mentioned.

In the present study, magnesium, calcium, iron, vitamin D and cobalamin were the most frequently supplemented nutrients. Cobalamin intake was strongly dependent on supplementation, especially for both female and male VEG. Half of the VEG group supplemented cobalamin and, thus, had a significantly higher intake compared to the D-A-CH reference values of 4 μg per day [33]. However, the very high cobalamin intake of the vegan supplement user can be classified as uncritical [56, 57]. In addition, the absorption rate decreases with increasing dosage. As expected, subjects of the VEG group who did not take cobalamin supplements had a marginal intake. Additionally, the dietary intake of the LOV group was insufficient, especially for males, who had cobalamin intakes below the recommendations, regardless of supplementation. However, although consuming cobalamin-rich foods such as meat, meat products and fish, its intake was still inadequate in one-third of the OMN group. Cobalamin is considered critical for VEG, but adequate intake should be ensured for every diet.

Due to high riboflavin levels in animal products, it was not surprising that the OMN group consumed the highest amounts, although, on average, VEG and female LOV reached the recommendations, which agrees with previous studies in non-athletes [58, 59]. In contrast to Eisinger and colleagues, who showed high intakes of riboflavin in LOV endurance runners [60], only female LOV achieved the reference values. Pyridoxine intake exceeded the recommendations in the VEG group due to the high consumption of vegetables, legumes, nuts, and seeds, which has already been shown by other studies with non-athletes [58, 61]. The VEG group showed a high folate intake due to the high amount of folate in green vegetables, yeast, and nuts, while the folate intake of most OMN subjects was insufficient. These results are consistent with the German Nutrition Survey [52] and studies with athletes [54].

Similar to cobalamin, vitamin D intake was strongly dependent on the use of supplements. This becomes clear by comparing the vitamin D intake between SU and non-SU. On average, the VEG group (19.9, 2.75–37.0 μg) was closest to the recommendations of 20 μg per day compared to OMN (8.29, 2.21–14.4 μg) and LOV (4.52, − 1.14–10.4 μg). However, the intake of vitamin D was considerably higher in SU compared to non-SU. Hence, the mean values for the vitamin D intake in the VEG group (including SU and non-SU) should be treated with caution. This also applies to the OMN and LOV group, although not quite as strongly pronounced. However, it is worth mentioning that an adequate vitamin D status can only be evaluated with the endogenous 25-hydroxyvitamin D status in the blood [62].

Similar to other studies with non-athletes [42, 58], the highest iron intake from food (excluding supplements) was observed in VEG subjects compared to LOV and OMN. In addition, the VEG group had the highest iron intake via supplements compared to the other two groups. A total of more than 85% of VEG subjects achieved the recommendations compared to only ~ 50% in OMN and LOV. Male subjects of all groups were above the recommendations with more than 10 mg per day, independent of supplementation. Female OMN and LOV subjects achieved the recommendation of 15 mg daily only after supplementation. Interestingly, the VEG group reached the iron intake recommendations solely via food and not via supplements. The literature on the iron supply of athletes is inconsistent. Some studies found an adequate [63, 64] and others an inadequate iron intake in athletes [65]. High-performance athletes might have increased requirements due to biochemical adaptations (e.g. increased blood formation and increased enzymatic antioxidant defense) and increased iron losses via sweat, urine, and feces, which results in a higher risk of iron deficiency anemia [64]. In addition to absolute amounts, the bioavailability of different iron species should be considered. Despite the exclusive consumption of plant-based iron of the VEG group, LOV and OMN also consumed predominantly plant iron sources. While plant-based foods contain non-heme iron, mainly in trivalent form (Fe^3+^), which has a poor bioavailability of 1–5%, meat and fish contain about 70% of the total iron in the form of heme iron, which can be absorbed much better at 10–20% [66, 67]. Hence, the lower iron intake in OMN subjects compared to LOV and VEG does not necessarily result in a lower status. Moreover, further influences on bioavailability (promoting substances such as ascorbic acid or lactic acid and inhibiting substances such as phytic acid or oxalic acid, which occurs in vegetable foods) must be taken into account (the same applies to zinc, magnesium, and calcium). Therefore, only functional parameters, such as transferrin and ferritin, indicate an adequate supply status.

The present results show that calcium is a critical nutrient [55]. As expected, calcium intake was highest among OMN subjects, although more than half were below the reference range. The highest number of subjects with an intake below the reference range for calcium was found in the LOV group (64.0%), although they consumed milk and dairy products. The athlete’s dietary intake of calcium should be improved independently of dietary habits due to the importance of bone health, and normal nerve and muscle function [68]. The mean intake of zinc was within the reference range for all groups, although male LOV subjects were slightly below. Female participants and non-SU of all three groups reached the recommendations [33]. Interestingly, the zinc supply was similar in OMN and VEG subjects, although animal-based foods are rich in zinc and the zinc supplement intake in the VEG group was considerably lower than OMN. These results reveal that zinc-rich plant-based foods can secure adequate zinc supply. The literature on zinc supply is inconsistent. Some studies observed a slightly lower but adequate intake of zinc in vegetarians and VEG compared to OMN [43, 48, 58], other studies found no differences between vegetarian and OMN endurance athletes [26].

The fact that the data of dietary intake relied on self-reported data by subjects should be considered. Both under- and over-reporting are further sources of error in dietary records. Since the use of iodized salt is voluntary in Germany and a precise indication about the dietary intake is critical, the values of iodine intake should be considered with caution. Furthermore, there are limitations regarding the nutrition software that shows data gaps, especially regarding vegan products. We did not consider the water intake of the subjects, which might also influence nutrient (e.g. mineral) supply.

## Conclusion

In summary, all three groups were adequately supplied with most nutrients. As expected, the intake of carbohydrates and fiber was highest in the VEG group, while the recommended amount of fat was not reached. Moreover, all three groups exceeded the recommendations for absolute protein intake. The mean intake of micronutrients was partly dependent on supplementation, especially for vitamin D and cobalamin. Only female VEG achieved the recommended amounts for iron intake solely via food and not via supplements. However, the demand for several micronutrients might be higher for athletes due to increased requirements and losses, especially when exogenous factors such heat occur [69–72]. Recommendations of current guidelines for adequate micronutrient intakes of ambitious recreational athletes are sparse due to a lack of data and future studies should clarify if specific recommendations are necessary.

## Supplementary information

**Additional file 1.** Dietary intake of essential amino acids (mg/kg BW) according to dietary pattern.

**Additional file 2.** Dietary intake of fatty acids according to dietary pattern.

**Additional file 3.** Proportion of participants who did not reach the recommended dietary intake of minerals and vitamins. Dietary intake is depicted in addition to supplement intake.

## Data Availability

The datasets used and/or analyzed during the current study are available from the corresponding author on reasonable request.

## Abbreviations

ACSM: American College of Sports Medicine
ALA: Alpha-linolenic acid
ANOVA: Analysis of variance
BMI: Body mass index
bpm: Beats per minute
BW: Body weight
CI: Confidence interval
D-A-CH: Deutsche, Österreichische und Schweizerische Gesellschaften für Ernährung, German, Austrian and Swiss Nutrition Societies
DHA: Docosahexaenoic acid
EN%: Energy percent
EPA: Eicosapentaenoic acid
IOC: International Olympic Committee
ISSN: International Society for Sports Nutrition
LA: Linoleic acid
LOV: Lacto-ovo vegetarians
n.s.: Not significant
non-SU: Non-supplement users
OMN: Omnivores
PUFA: Polyunsaturated fatty acid
SD: Standard deviation
SU: Supplement users
VEG: Vegans
WHO: World Health Organization
