# Supplementary material for: Characterization, dietary habits and nutritional intake of omnivorous, lacto-ovo vegetarian and vegan runners – a pilot study
Source: BMC Nutr. 2019 Dec 3;5:51. doi: 10.1186/s40795-019-0313-8 (PMC7050782; doi:10.1186/s40795-019-0313-8)
Supplement: Supplementary file 1 — Additional file 1. Dietary intake of essential amino acids (mg/kg BW) according to dietary pattern. [file 40795_2019_313_MOESM1_ESM.docx]

Additional file 1 Dietary intake of essential amino acids (mg/kg BW) according to dietary pattern.

|  |  |  |  |  |  |  |  |  |
| --- | --- | --- | --- | --- | --- | --- | --- | --- |
| **Amino acid** | **OMN**  **(n=27)** | **P value**  **OMN-LOV** | **LOV**  **(n=26)** | **P value**  **LOV-VEG** | **VEG**  **(n=28)** | **P value**  **OMN-VEG** | **P value**  **3 groups** | **Reference values (m/f)*** |
|  |  |  |  |  |  |  |  |  |
|  |  |  |  |  |  |  |  |  |
| **Isoleucine** | 54.9 (48.1, 61.6) | n.s. | 50.0 (32.7, 67,2) | n.s. | 41.0 (32.4, 50.0) | 0.008 | **0.007^a^** | 20 |
| **Leucine** | 92.0 (80.8, 103) | 0.047 | 83.2 (55.1, 111) | n.s. | 68.9 (54.8, 83.0) | 0.012 | **0.008^a^** | 39 |
| **Lysine** | 75.8 (64.7, 86.9) | 0.007 | 58.8 (36.7, 80.8) | n.s. | 44.1 (35.4, 52.9) | 0.000 | **0.000^a^** | 30 |
| **Methionine** | 25.8 (21.9, 29.6) | 0.015 | 20.5 (12.6, 28.4) | n.s. | 15.2 (11.6, 18.8) | 0.000 | **0.000^a^** | 10 |
| **Phenylalanine** | 52.8 (47.2, 58.5) | n.s. | 48.6 (33.6, 63.7) | n.s. | 44.4 (34.6, 54.2) | 0.044 | **0.022^a^** | 25 |
| **Threonine** | 46.2 (40.2, 52.1) | 0.020 | 38.7 (26.0, 51.4) | n.s. | 34.2 (27.6, 40.9) | 0.015 | **0.006^a^** | 15 |
| **Tryptophan** | 12.8 (11.2, 14.4) | n.s. | 11.5 (7.90, 15.2) | n.s. | 10.9 (8.70, 13.1) | n.s. | **0.037^a^** | 4 |
| **Valine** | 64.2 (56.6, 71.7) | n.s. | 58.0 (39.2, 76.8) | n.s. | 48.7 (39.0, 58.5) | 0.012 | **0.009^a^** | 26 |
| **Histidine** | 31.7 (27.8, 35.6) | 0.011 | 26.0 (17.5, 34.5) | n.s. | 22.9 (18.6, 27.2) | 0.009 | **0.003^a^** | 10 |
|  |  |  |  |  |  |  |  |  |

OMN = omnivores, LOV = lacto-ovo-vegetarians, VEG = vegans, * reference values of the World Health Organization [1].

Data are presented as mean (95% KI). ^a^ Kruskal Wallis test, ^b^ Post Hoc Test.
