# Supplementary material for: Characterization, dietary habits and nutritional intake of omnivorous, lacto-ovo vegetarian and vegan runners – a pilot study
Source: BMC Nutr. 2019 Dec 3;5:51. doi: 10.1186/s40795-019-0313-8 (PMC7050782; doi:10.1186/s40795-019-0313-8)
Supplement: Supplementary file 2 — Additional file 2. Dietary intake of fatty acids according to dietary pattern. [file 40795_2019_313_MOESM2_ESM.docx]

Additional file 2 Dietary intake of fatty acids according to dietary pattern.

|  |  |  |  |  |  |  |  |  |
| --- | --- | --- | --- | --- | --- | --- | --- | --- |
| **Fatty acid** | **OMN**  **(n=27)** | **P value**  **OMN-LOV** | **LOV**  **(n=26)** | **P value**  **LOV-VEG** | **VEG**  **(n=28)** | **P value**  **OMN-VEG** | **P value**  **3 groups** | **Reference values (m/f)*** |
|  |  |  |  |  |  |  |  |  |
|  |  |  |  |  |  |  |  |  |
| **SFA (EN%)** | 8.70 (7.13, 10.3) | n.s. | 7.86 (6.17, 9.55) | 0.006^b^ | 4.57 (3.55, 5.59) | 0.000^b^ | **0.000^a^** | 7-10 |
| **MUFA (EN%)** | 5.95 (4.86, 7.03) | n.s. | 5.45 (3.77, 7.13) | n.s. | 3.96 (3.02, 4.91) | 0.019^b^ | **0.024^a^** | > 10 |
| **PUFA (EN%)** | 2.81 (2.29, 3.32) | - | 3.21 (2.14, 2.97) | - | 3.39 (2.63, 4.14) | - | 0.513^a^ | 7-10 |
| **EPA (g)** food  supplement | 0.19 (0.32, 0.35)  0.08 (-0.03, 0.19) | 0.000^b^  - | 0.01 (0.00, 0.01)  0.01 (-0.01, 0.03) | n.s.  - | 0.00 (0.00, 0.00)  0.04 (-0.03, 0.10) | 0.000^b^  - | **0.000^a^**  0.823^a^ | - |
| **DHA (g)** food  supplement | 0.25 (0.14, 0.35)  0.03 (-0.02, 0.08) | 0.031^b^  - | 0.06 (0.03, 0.87)  0.01 (-0.01, 0.02) | n.s.  - | 0.03 (0.01, 0,06)  0.01 (-0.01, 0.04) | 0.000^b^  - | **0.000^a^**  0.821^a^ | - |
| **LA (EN%)** | 2.96 (2.50, 3.42) | n.s. | 3.52 (2.57, 4.46) | n.s. | 4.33 (3.44, 5.21) | n.s. | **0.049^a^** | 2.5 |
| **ALA (EN%)** | 0.37 (0.27, 0.48) | n.s. | 0.68 (0.33, 1.03) | n.s. | 0.80 (0.55, 1.05) | 0.005^b^ | **0.007^a^** | 0.5 |
| **LA:ALA ratio** | 1:8.04 | - | 1:5.30 | - | 1:5.71 | - | 0.481^a^ | 1:5 |
|  |  |  |  |  |  |  |  |  |
| OMN = omnivores, LOV = lacto-ovo-vegetarians, VEG = vegans, SFA = saturated fatty acids, PUFA = polyunsaturated fatty acids, MUFA = monounsaturated fatty acids, EPA = eicosapentaenoic acid, DHA = docosahexaenoic acid, ALA= alpha linolenic acid, LA= linoleic acid, EN% = energy percent, n.s. = not significant, * reference values of the German, Austrian and Swiss Nutrition Societies (Deutsche, Österreichische und Schweizerische Gesellschaften für Ernährung, D-A-C-H) [2].  Data are presented as mean (95% KI). ^a^ Kruskal Wallis test, ^b^ Post Hoc Test. | | | | | | | | |
