# Supplementary material for: Characterization, dietary habits and nutritional intake of omnivorous, lacto-ovo vegetarian and vegan runners – a pilot study
Source: BMC Nutr. 2019 Dec 3;5:51. doi: 10.1186/s40795-019-0313-8 (PMC7050782; doi:10.1186/s40795-019-0313-8)
Supplement: Supplementary file 3 — Additional file 3. Proportion of participants who did not reach the recommended dietary intake of minerals and vitamins. Dietary intake is depicted in addition to supplement intake. [file 40795_2019_313_MOESM3_ESM.docx]

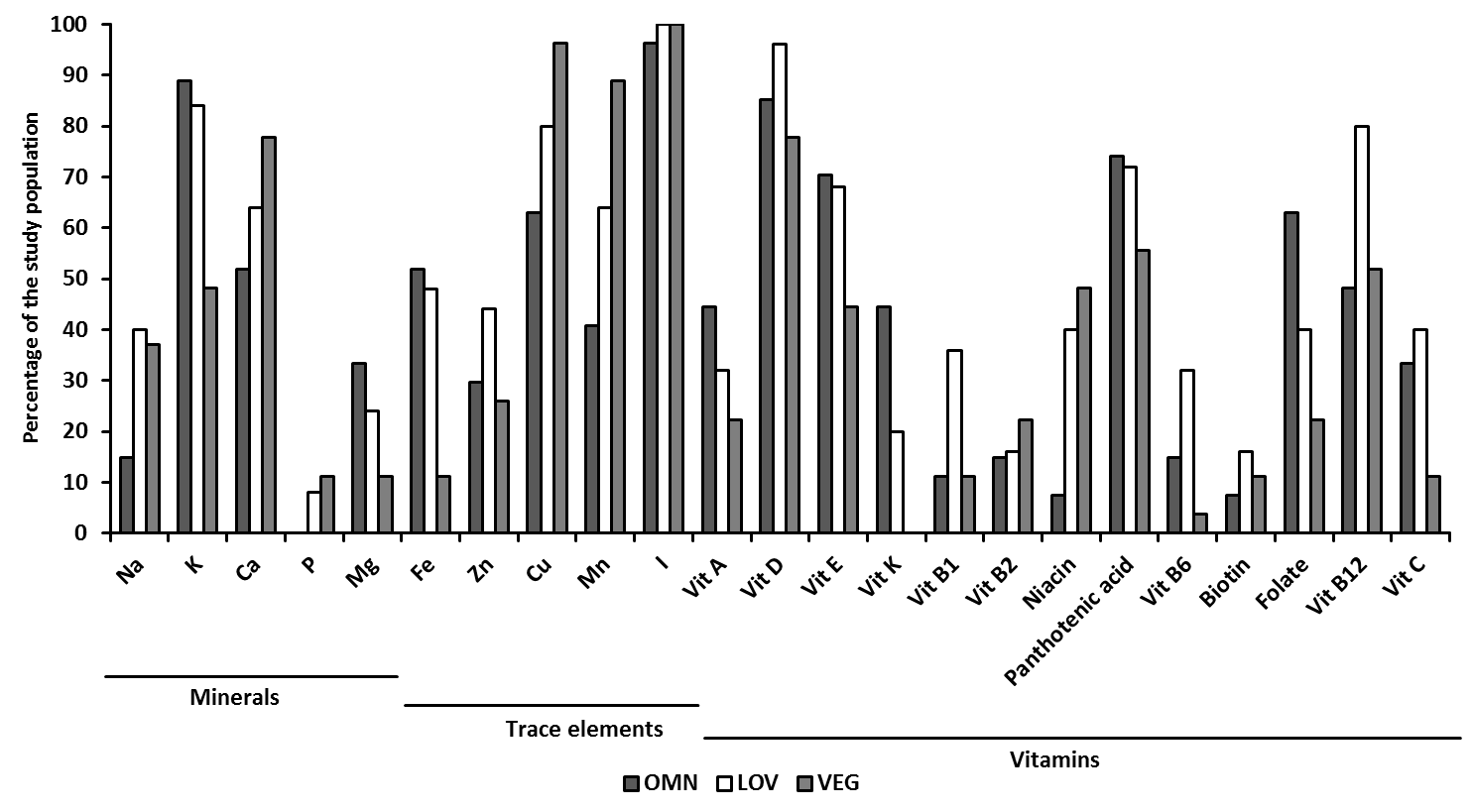


**Additional file 3** Proportion of participants who did not reach the recommended dietary intake of minerals and vitamins. Dietary intake is depicted in addition to supplement intake. OMN = omnivores, LOV = lacto-ovo-vegetarians, VEG = vegans, Vit = vitamin
